# A Twin Study on the Association Between Psychotic Experiences and Tobacco Use During Adolescence

**DOI:** 10.1016/j.jaac.2018.06.037

**Published:** 2019-02

**Authors:** Wikus Barkhuizen, Mark J. Taylor, Daniel Freeman, Angelica Ronald

**Affiliations:** a Centre for Brain and Cognitive Development, University of London, UK; b Karolinska Institutet, Stockholm, Sweden; c University of Oxford, UK

**Keywords:** psychotic-like experiences, youth, smoking, cigarette, heritability

## Abstract

**Objective:**

Psychotic experiences (PE) are dimensional phenomena in the general population that resemble psychotic symptoms, such as paranoia and hallucinations. This is the first twin study to explore the degree to which tobacco use and PE share genetic or environmental influences. Previous studies on the association between adolescent tobacco use and PE have not considered PE dimensionally, included negative symptoms, or accounted for confounding by sleep disturbance and stressful life events.

**Method:**

An unselected adolescent twin sample (N = 3,787 pairs; mean age = 16.16 years) reported on PE (paranoia, hallucinations, cognitive disorganization, grandiosity, and anhedonia) and regularity of tobacco use. Parents rated the twins’ negative symptoms. Regression analyses were conducted while adjusted for sociodemographic characteristics, prenatal maternal smoking, cannabis use, sleep disturbance, and stressful life events. Bivariate twin modeling was used to estimate the degree of genetic and common and unique environmental influences shared between tobacco use and PE.

**Results:**

Regular smokers were significantly more likely to experience paranoia, hallucinations, cognitive disorganization, and negative symptoms (β = 0.17−0.34), but not grandiosity or anhedonia, than nonsmokers, after adjustment for confounders. Paranoia, hallucinations, and cognitive disorganization correlated ≥0.15 with tobacco use (*r* = 0.15−0.21, all *p* < .001). Significant genetic correlations (*r*_A_=0.37−0.45) were found. Genetic influences accounted for most of the association between tobacco use and paranoia (84%) and cognitive disorganization (81%). Familial influences accounted for 80% of the association between tobacco use and hallucinations.

**Conclusion:**

Tobacco use and PE during adolescence were associated after adjustment for confounders. They appear to co-occur largely because of shared genetic influences.

Tobacco use, a modifiable behavior, is associated with psychotic experiences (PE).^1^, ^2^, ^3^, ^4^, ^5^, ^6^, ^7^, ^8^, ^9^ PE are common dimensional phenomena in the general population that range in severity and resemble psychotic symptoms at the extreme, such as paranoia and hallucinations. Those who experience PE, especially if they persist, are at higher risk for developing psychotic and other psychiatric disorders.^10^, ^11^, ^12^, ^13^, ^14^, ^15^ Longitudinal evidence that adolescent tobacco use precedes PE suggests a possible role of smoking in the development of PE.^3^, ^8^ In the United Kingdom, 35% of 15-year-olds have smoked cigarettes at least once,^16^ and in the United States, 21% of 15- to 17-year-olds,^17^ with 3% to 5.5% smoking daily.^18^ Understanding the nature of the association between adolescent PE and tobacco use is therefore important. There is a lack of evidence regarding whether PE and tobacco use share genetic or environmental influences. Furthermore, although previous studies considered confounders such as cannabis use and sociodemographic characteristics on the association between PE and smoking, factors such as sleep disturbance and stressful life events have been ignored.

Previous studies on tobacco use and PE, mostly conducted on adults,^1^, ^2^, ^4^, ^6^, ^7^ found that regular tobacco use increased the odds of experiencing PE by 20% to 47% after accounting for sociodemographic characteristics, cannabis use, and comorbid mental and physical disorders. These studies measured only positive PE, such as hallucinations and delusions, defined categorically. In the studies that assessed age of smoking initiation, individuals who started smoking during or prior to adolescence were more likely to report PE as adults.^2^, ^6^

Other factors associated with PE during adolescence include cannabis use,^9^, ^19^, ^20^, ^21^ stressful life events,^22^, ^23^ illicit drug use,^3^, ^14^ sleep disturbance,^24^, ^25^ and prenatal maternal smoking.^26^ Because these factors may also be associated with tobacco use, it is sensible to investigate the contribution of tobacco use while accounting for these factors, some of which were not considered previously.

Although tobacco might be considered an environmental exposure, twin studies estimate significant heritability of tobacco use during adolescence (36%−60%).^27^, ^28^, ^29^, ^30^, ^31^ It is therefore important to consider both genetic and environmental influences when investigating the association between adolescent PE and tobacco use. Twin studies on PE indicate genetic and unique environmental influences as important; common environmental influences are small or negligible.^32^

We aimed to investigate whether associations between tobacco use and PE exist in adolescence and whether they remain after adjusting for sex, age, ethnicity, socio-economic status, cannabis use, stressful life events, sleep disturbance, and prenatal maternal smoking. Our second aim was to test whether shared genetic and environmental influences underlie adolescent PE and tobacco smoking. We consider a range of PE including paranoia, hallucinations, cognitive disorganization, grandiosity, anhedonia, and parent-rated negative symptoms (such as flattened affect and low motivation), assessed as dimensional traits.

## Method

### Sample

Research participants came from the Twins Early Development Study (TEDS), a UK-based community sample of twins born between 1994 and 1996^33^ who, along with their parents, participated in the Longitudinal Experiences and Perceptions Study (LEAP)^34^ at age 16 years. Of the 8,534 families invited to report on PE and substance use, 3,941 (46.2%) participated. Table S1, available online, compares participating and nonparticipating families.

For regression analyses, one twin per family was chosen randomly to select a sample of unrelated individuals. After exclusions (missing zygosity data, no consent information, severe medical conditions, or perinatal complications), the sample included 3,787 individuals.

For twin analyses, zygosity was determined using a parent-rated measure and confirmed using DNA testing in ambivalent cases. The sample, after exclusions, consisted of 1,342 monozygotic (MZ; 43.8% male) and 1,219 same-sex dizygotic (DZ) pairs (44.5% male). Opposite-sex DZ pairs (1,208) were not included because of limited power in bivariate twin models to test for qualitative sex differences in categorical data.

The Institute of Psychiatry Ethics Committee, King’s College London, granted ethical approval for TEDS. All research participants granted informed consent. The Birkbeck Department of Psychological Sciences’ Ethics Committee and the core TEDS team approved this study.

### Measures

#### Psychotic Experiences

The Specific Psychotic Experiences Questionnaire (SPEQ)^34^ included three subscales measuring “positive” PE (paranoia, hallucinations, and grandiosity), two measuring “negative” PE (parent-rated negative symptoms and anhedonia), and one cognitive disorganization subscale. Subscales consisted of eight to 15 items and asked about frequency or severity of recent PE. Subscales showed good-to-excellent internal consistency (Cronbach’s α = 0.77–0.93) and test–retest reliability over 9 months (*r* = 0.65–0.74). Negative symptoms were parent-rated; other subscales were self-rated.

Untransformed SPEQ scores were used in descriptive statistics. Subscales with a positive skew (paranoia, hallucinations, grandiosity, and parent-rated negative symptoms) were square root transformed. PE were standardized to have a mean of 0 and a standard deviation (SD) of 1.

#### Tobacco Use

A tobacco use variable was created with three levels. Nonsmokers were those who answered “no” to the question “Have you ever smoked a cigarette (including roll-ups)?” Occasional and regular smokers were defined based on the item “How many cigarettes have you smoked, in total, in your lifetime?” Occasional smokers were those who had smoked <50 cigarettes and regular smokers ≥50. Those who had not smoked in the past year were considered nonsmokers. The threshold of 50 cigarettes (rather than a higher threshold) was chosen because adolescents have had less access to resources and opportunity to smoke than adults. For smoking frequency by group, see Table S2, available online.

#### Confounding Variables

Variables adjusted for during multiple regression included sex, age, ethnicity, socioeconomic status (SES), prenatal maternal smoking, and self-rated measures (at age 16 years) of cannabis use, sleep disturbance measured by the Pittsburgh Sleep Quality Index (PSQI),^35^ and stressful life events (SLE) measured by an abbreviated Coddington Life Events Record.^36^

Cannabis use was determined from “yes” responses to “Have you ever tried cannabis?” Those who indicated no use during the past year were considered non−cannabis-users. SES was a standardized score derived from parent qualifications and employment and mother’s age at birth of her first child (ascertained at first contact when the twins were aged 18 months). Prenatal maternal smoking was determined at first contact from “yes” responses to “Did you smoke cigarettes while pregnant?” SLE scores were square root transformed to reduce skewness. Variables were standardized for regression analyses.

### Data Analyses

Differences among nonsmokers, occasional smokers, and regular smokers on SPEQ scores and covariates were tested using one-way analyses of variance (for continuous variables) and χ^2^ tests (categorical variables). Pairwise comparisons were performed using Bonferroni-corrected *p* values.

#### Regression Analyses

Regression analyses were conducted using the *lm()* function in R.^37^ PE were the outcome variables and tobacco use the exposure. Missing data were omitted on a pairwise basis. Linear regression between PE and tobacco use was run to estimate unadjusted models. In adjusted models, predictor variables were entered simultaneously. The presence of multicollinearity was assessed by computing variance inflation factors (VIF), where VIF > 4 indicates multicollinearity. Significance thresholds were set at *p* < .05 and adjusted *R*^2^ values reported to account for the number of predictors in models.

#### Structural Equation Twin Modeling

The twin design enables individual differences to be decomposed into genetic and environmental components. If a trait is heritable, MZ within-pair similarity is higher than DZ similarity. Shared environmental influences make children growing up in the same family similar as indexed by DZ within-pair similarity being greater than half MZ within-pair similarity. Nonshared environmental influences make children growing up in the same family different, present where MZ within-pair similarity is less than unity.

Structural equation twin modeling was conducted in OpenMx 2.0^38^ for R^37^. The effects of sex and age were regressed out of SPEQ scales after normalization. Twin models were fitted if the correlation between PE and tobacco use was >0.15 to allow for enough covariance to be decomposed into genetic and environmental influences. Liability-threshold models, which assume an underlying normal distribution to ordinal data, were fitted for univariate tobacco use models. For bivariate analyses, joint ordinal−continuous twin models were fitted that modeled liability to tobacco use and variation in PE.

Twin correlations were calculated using intraclass correlations. Saturated models, constraining means, thresholds, and phenotypic correlations across twin order, were run between PE and tobacco use to calculate cross-twin cross-trait (CTCT) and phenotypic polyserial correlations. Saturated models provide a full description of the data prior to decomposing variance/covariance into genetic (A), common environmental (C), and unique environmental (E) influences. ACE models were compared to saturated models. Subsequently, statistical significance of the variance components was tested by systematically fixing each to zero in submodels (while retaining E because it contains residual error) and comparing to ACE models. The most parsimonious model is preferred and was identified if a likelihood-ratio test at *p* < .05 indicated a not significantly worse fit compared to full models and based on the lowest Akaike Information Criterion (AIC).

The extent to which the same genes or environments influence PE and tobacco use was estimated from the genetic correlations (r_A_), shared environmental correlations (r_C_), and unique environmental correlations (r_E_). The bivariate heritability (biva^2^) and equivalent bivariate values for shared (bivc^2^) and unique environments (bive^2^) provided estimates of the degree of covariation between tobacco use and PE explained by A, C, or E. These values were divided by the phenotypic correlations to calculate the proportion of the covariance explained by genetic or environmental influences.

## Results

In the current sample (N = 3,787; 45.9% male; mean 16.16 years, SD = 0.68), 31.4% reported ever having smoked cigarettes. Of the 3,610 adolescents who provided information on the regularity of their tobacco use in the past year, 2,985 (82.7%) were nonsmokers, 436 (12.1%) occasional smokers, and 189 (5.2%) regular smokers.

The PE scores were significantly different across tobacco use groups (Table 1). Pairwise comparisons indicated that occasional smokers scored significantly higher than nonsmokers on paranoia (*p* < .001), hallucinations (*p* = .002), and cognitive disorganization (*p* < .001) and lower on anhedonia (*p* = .023), with no significant differences on grandiosity (*p* = 1.00) and parent-rated negative symptoms (*p* = .078). Compared to nonsmokers, regular smokers scored significantly higher on paranoia (*p* < .001), hallucinations (*p* < .001), cognitive disorganization (*p* < .001), grandiosity (*p* = .002), and negative symptoms (*p* < .001), but not on anhedonia (*p* = .061). Compared to occasional smokers, regular smokers scored significantly higher on all six scales including paranoia (*p* = .006), hallucinations (*p* < .001), cognitive disorganization (*p* < .001), grandiosity (*p* = .018), anhedonia (*p* = .001), and negative symptoms (*p* < .001).

**Table 1:** Sample Characteristics and Descriptive Statistics for Total Sample and Split by Tobacco Use

Characteristic	Range	Total Sample		Nonsmokers		Occasional Smokers		Regular Smokers		*F*	*p*
		n	Mean (SD)	n	Mean (SD)	n	Mean (SD)	n	Mean (SD)		
**Psychotic experiences (SPEQ)**											
Paranoia	0–75	3,603	11.70 (10.54)	2,978	11.12 (10.31)	436	13.43 (10.56)	189	16.25 (12.81)	30.33	<.001
Hallucinations	0–45	3,607	4.56 (6.07)	2,983	4.28 (5.81)	436	5.33 (6.62)	188	7.35 (8.16)	25.61	<.001
Cognitive disorganization	0–11	3,606	3.90 (2.83)	2,982	3.72 (2.79)	436	4.27 (2.75)	188	5.64 (2.95)	46.86	<.001
Grandiosity	0–24	3,606	5.41 (4.52)	2,981	5.34 (4.47)	436	5.39 (4.28)	189	6.47 (5.32)	4.52	.011
Anhedonia	0–50	3,604	16.31 (7.79)	2,979	16.36 (7.84)	436	15.29 (7.14)	189	17.71 (8.58)	6.86	.001
Negative symptoms	0–30	3,583	2.86 (3.88)	2,965	2.74 (3.69)	431	2.52 (3.64)	187	4.57 (5.26)	19.49	<.001
**Covariates (continuous)**											
Age	14.9–18.7	3,610	16.16 (.68)	2,985	16.12 (.68)	436	16.35 (.65)	189	16.38 (.62)	33.44	<.001
SES	–2.6 to 2.6	3,430	0.25 (.98)	2,840	0.26 (.97)	412	.36 (1.02)	178	−0.07 (1.01)	12.06	<.001
Sleep disruption	0–21	3,603	5.48 (2.69)	2,980	5.32 (2.64)	434	5.92 (2.79)	189	6.72 (3.04)	31.52	<.001
Stressful life events	0–20	3,236	2.31 (1.74)	2,672	2.12 (1.61)	392	2.79 (1.72)	172	3.92 (2.42)	79.98	<.001

**Covariates (categorical)**		**n**		**n**	**%**	**n**	**%**	**n**	**%**	**χ**^**2**^	***p***
Sex										0.07	.967
Males		1,660		1,370	82.53%	203	12.23%	87	5.24%		
Females		1,950		1,615	82.82%	233	11.95%	102	5.23%		
Ethnicity										8.31	.016
White		3,376		2,778	82.29%	420	12.44%	178	5.27%		
Other		225		201	89.33%	14	6.22%	10	4.44%		
Cannabis use										1099.90	<.001
No		3,159		2,666	84.39%	372	11.78%	121	3.83%		
Yes		438		307	70.09%	63	14.38%	68	15.53%		
Maternal smoking during pregnancy										111.81	<.001
No		3,159		2,666	84.39%	372	11.78%	121	3.83%		
Yes		438		307	70.09%	63	14.38%	68	15.53%		

Note: SPEQ = Specific Psychotic Experiences Questionnaire.

### Regression Models

The VIF for all predictors ranged between 1.07 and 1.51, which indicated no multicollinearity between predictor variables (see Table S3, available online, for correlations between variables). Unadjusted models (Table 2) indicated that regular smoking, compared to not smoking, significantly (*p* < .05) predicted higher scores on all PE subscales. Occasional smoking, compared to not smoking, significantly predicted higher scores for paranoia, hallucinations, and cognitive disorganization and lower scores for anhedonia, and did not predict grandiosity and parent-rated negative symptoms. Models accounted for 0.3% to 2.5% of variance in PE scores, most for cognitive disorganization, paranoia, and hallucinations.

**Table 2:** Linear Regression Models Showing Tobacco Use as a Predictor of Psychotic Experiences

	Unadjusted Model			Adjusted Model				Unadjusted Model			Adjusted Model		
	*R*^2^	*β*	95% CI	*R*^2^	*β*	95% CI		*R*^2^	*β*	95% CI	*R*^2^	*β*	95% CI
**Paranoia**	.017			.160			**Grandiosity**	.003			.046		
Tobacco use							Tobacco use						
Occasional smokers		**0.27**	**0.17–0.37**		**0.13**	**0.02–0.23**	Occasional smokers		0.05	–0.05–0.15		0.05	–0.07–0.17
Regular smokers		**0.46**	**0.31–0.60**		**0.17**	**0.00–0.34**	Regular smokers		**0.22**	**0.07–0.37**		0.17	–0.01–0.35
Sex					–0.04	–0.11–0.02	Sex					**0.26**	**0.19–0.33**
Age					**–0.08**	**–0.13 to –0.03**	Age					–0.02	–0.08–0.03
Ethnicity					–0.07	–0.21–0.07	Ethnicity					**0.36**	**0.21–0.51**
Cannabis use					0.10	–0.04–0.24	Cannabis use					–0.04	–0.19–0.12
Sleep disturbance					**0.34**	**0.31–0.38**	Sleep disturbance					–0.02	–0.05–0.02
Significant life events					**0.10**	**0.07–0.14**	Significant life events					**0.15**	**0.12–0.19**
Socio-economic status					**0.08**	**0.05–0.12**	Socio-economic status					0.01	–0.02–0.05
Prenatal maternal smoking					0.01	–0.10–0.11	Prenatal maternal smoking					0.00	–0.12–0.11
**Hallucinations**	0.014			.145			**Anhedonia**	.004			.083		
Tobacco use							Tobacco use						
Occasional smokers		**0.19**	**0.09–0.29**		0.07	–0.04–0.18	Occasional smokers		**–0.14**	**–0.24 to –0.04**		**–0.12**	**–0.23 to –0.01**
Regular smokers		**0.48**	**0.33–0.62**		**0.24**	**0.06–0.41**	Regular smokers		**0.17**	**0.03–0.32**		0.16	–0.02**–**0.34
Sex					–**0.10**	**–0.16 to** –**0.03**	Sex					**0.49**	**0.42–0.56**
Age					–**0.09**	**–0.14 to –0.04**	Age					–0.01	–0.06–0.04
Ethnicity					0.11	–0.03–0.26	Ethnicity					0.00	–0.15–0.15
Cannabis use					0.11	–0.04–0.25	Cannabis use					0.01	–0.14–0.16
Sleep disturbance					**0.32**	**0.28–0.35**	Sleep disturbance					**0.14**	**0.11–0.18**
Significant life events					**0.12**	**0.08–0.15**	Significant life events					**–0.11**	**–0.15 to –0.07**
Socio-economic status					–0.03	–0.07–0.00	Socio-economic status					0.02	–0.02–0.06
Prenatal maternal smoking					–0.03	–0.14–0.07	Prenatal maternal smoking					0.01	–0.11–0.12
**Cognitive disorganization**	0.025			0.226			**Negative symptoms**^a^	.011			.052		
Tobacco use							Tobacco use						
Occasional smokers		**0.20**	**0.10–0.30**		0.09	–0.02–0.19	Occasional smokers		–0.06	–0.16–0.04		–0.06	–0.18–0.05
Regular smokers		**0.68**	**0.54–0.83**		**0.34**	**0.17–0.50**	Regular smokers		**0.45**	**0.30–0.59**		**0.23**	**0.04–0.41**
Sex					**–0.30**	**–0.37 to –0.24**	Sex					**0.19**	**0.12–0.26**
Age					**–0.05**	**–10 to –0.00**	Age					**–0.10**	**–0.15 to –0.04**
Ethnicity					0.02	–0.11–0.16	Ethnicity					0.06	–0.09–0.21
Cannabis use					0.06	–0.08–0.20	Cannabis use					0.05	–0.10–0.21
Sleep disturbance					**0.40**	**0.36–0.43**	Sleep disturbance					**0.14**	**0.10–0.18**
Significant life events					**0.06**	**0.02–0.09**	Significant life events					–0.02	–0.06–0.02
Socio-economic status					**–0.08**	**–0.11 to –0.04**	Socio-economic status					**–0.13**	**–0.17 to –0.09**
Prenatal maternal smoking					0.03	–0.08–0.13	Prenatal maternal smoking					**0.13**	**0.01–0.24**

Note: Significance at *p* < .05 is shown in boldface type. Adjusted *R*^2^ reported for adjusted models. Reference groups: for tobacco use, non-smokers; sex, female; for ethnicity, white; for cannabis use, no; for prenatal maternal smoking, no. Unadjusted model results: paranoia (*F*_2,3600_ = 30.33, *p* < .001), hallucinations (*F*_2,3604_ = 25.61, *p* < .001), cognitive disorganization (*F*_2,3603_ = 46.86, *p* < .001), grandiosity (*F*_2,3602_ = 4.52, *p* = .011), anhedonia (*F*_2,3601_ = 6.86, *p* = .001) and parent-rated negative symptoms (*F*_2,3580_ = 19.49, *p* < .001). Adjusted model results: paranoia (*F*_10,2985_ = 57.88, *p* < .001), hallucinations (*F*_10,2989_ = 51.86, *p* < .001), cognitive disorganization (*F*_10,2989_ = 88.65, *p* < .001), grandiosity (*F*_10,2989_ = 15.56, *p* < .001), anhedonia (*F*_10,2986_ = 28.02, *p* < .001), and parent-rated negative symptoms (*F*_10,2970_ = 17.30, *p* < .001).

a Parent-rated.

Adjusted models accounted for 4.6% to 22.6% of variance in PEs, the highest being for cognitive disorganization (22.6%), paranoia (16%), and hallucinations (14.5%). Standardized coefficients indicated that paranoia increased by 0.13 and 0.17 SD in occasional and regular smokers, respectively, compared to nonsmokers. Models for hallucinations, cognitive disorganization, and parent-rated negative symptoms indicated an increase of 0.24, 0.34, and 0.23 SD, respectively, in regular smokers compared to nonsmokers; occasional smoking did not significantly predict these PE. Regular tobacco use did not significantly predict grandiosity in adjusted models. Anhedonia scores decreased on average by 0.12 SD in occasional smokers compared to nonsmokers. Compared to unadjusted models, standardized coefficients for tobacco use generally decreased in adjusted models. Sensitivity analyses using generalized estimating equation models to include both twins did not affect our conclusions (see Table S4, available online).

### Genetic and Environmental Influences on Psychotic Experiences and Tobacco Use

Phenotypic correlations (Table 3) were sufficiently large (>0.15) to run bivariate models between tobacco use and paranoia, hallucinations, and cognitive disorganization.

**Table 3:** Phenotypic Correlations and Univariate and Bivariate Twin Correlations

	Tobacco			
Phenotypic correlations	*r*	CI		
Paranoia	0.19	0.15–0.24		
Hallucinations	0.15	0.11–0.20		
Cognitive disorganization	0.21	0.16–0.25		
Grandiosity and delusions	0.01	–0.03–0.05		
Negative symptoms^a^	0.10	0.06–0.15		
Anhedonia	0.05	0.01–0.09		
	**MZ**		**DZ**	
Twin correlations	*r*	CI	*R*	CI
Paranoia	0.53	0.49–0.56	0.30	0.25–0.35
Hallucinations	0.43	0.38–0.47	0.29	0.24–0.34
Cognitive disorganization	0.46	0.41–0.50	0.22	0.17–0.28
Tobacco	0.82	0.76–0.87	0.68	0.60–0.75
Cross-twin cross-trait correlations (psychotic experiences and tobacco)				
Paranoia	0.15	0.10–0.20	0.12	0.06–0.17
Hallucinations	0.11	0.06–0.16	0.08	0.03–0.14
Cognitive disorganization	0.18	0.13–0.22	0.11	0.05–0.16

Note: DZ = dizygotic twins; MZ = monozygotic twins.

a Parent-rated.

Univariate MZ twin correlations (Table 3) were higher than DZ correlations for tobacco use, paranoia, hallucinations, and cognitive disorganization, which implied genetic influences (A). Common environmental influences (C) were indicated for hallucinations and tobacco use, and somewhat for paranoia, because DZ correlations were greater than half the MZ correlations, but not for cognitive disorganization. Unique environmental influences (E) were indicated for all measures because MZ correlations were <1.

A full ACE liability-threshold model was indicated for tobacco use (see Table S5, available online) with A = 0.32 (CI = 0.17−0.49), C = 0.51 (0.36−0.64), and E = 0.17 (0.13−0.29). A third of the variance in tobacco use was explained by genetic influences and half due to common environment. Univariate models for SPEQ subscales have been published previously from the TEDS sample^21^ showing genetic (A = 0.27−0.54) and nonshared environmental (E = 0.12−0.50) influences explain most variation in PE.

Cross-twin cross-trait (CTCT) correlations (Table 3) were higher in MZ than in DZ pairs, indicating A on the covariance between PE and tobacco use. The DZ CTCT correlations were greater than half those of MZ correlations for paranoia and hallucinations (but not for cognitive disorganization), implicating C influences on covariation. Some E influences on the covariation between tobacco use with paranoia, hallucinations, and cognitive disorganization were suggested, as MZ CTCT correlations were lower than phenotypic correlations.

Fit statistics for bivariate models (Table 4) indicated that, compared to saturated models, ACE models did not fit significantly worse. Partial AE models, in which C parameters were dropped for PE and for covariance paths between tobacco and PE (C was retained for tobacco because univariate results indicated that C influences tobacco use), were compared to the full ACE models. Partial AE models did not fit significantly worse than ACE models for tobacco use with both paranoia and cognitive disorganization. Partial AE models dropping genetic correlations had significantly worse fits compared to ACE models.

**Table 4:** Fit Statistics for Bivariate Twin Models of Tobacco Use and Psychotic Experiences

	Model	Base	Model Fit							Bivariate Statistics From Most Parsimonious Models (95% CI)					
			EP	−2LL	df	AIC	Δ−2LL	Δ df	*p*	Biva^2^	Bivc^2^	Bive^2^	r_A_	r_C_	r_E_
Paranoia	Saturated	–	13	18583.63	9990	–1396.37	–	–	–						
	ACE	Sat	12	18585.27	9993	–1400.73	1.64	3	.650						
	AE (retained C for tobacco)^a^	ACE	10	18588.47	9995	–1401.53	3.20	2	.202	0.16 (0.11–0.21)	–	0.03 (–0.01–0.06)	0.37 (0.25–0.53)	–	0.11 (–0.01–0.21)
	ACE dropped r_A_	ACE	11	18587.60	9994	–1400.40	2.33	1	.127						
	ACE dropped r_C_	ACE	11	18587.21	9994	–1400.79	1.94	1	.164						
	ACE dropped r_A_ and r_C_	ACE	10	18626.77	9995	–1363.23	41.50	2	<.001						
	AE dropped r_A_	ACE	9	18628.58	9996	–1363.42	43.31	3	<.001						
Hallucinations	Saturated	–	13	18696.66	9960	–1223.34	–	–	–						
	ACE^a^	Sat	12	18698.42	9963	–1227.58	1.76	3	.624	0.07 (–0.05–0.19)	0.05 (–0.05–0.15)	0.04 (0.01–0.07)	0.25 (–0.17–0.67)	0.16 (NA–0.51)	0.12 (0.01–0.23)
	AE (retained C for tobacco)	ACE	10	18706.94	9965	–1223.06	8.52	2	.014						
	ACE dropped r_A_^b^	ACE	11	18699.63	9964	–1228.37	1.21	1	.272	–	0.10 (0.06–0.14)	0.05 (0.02–0.08)	–	0.34 (0.19–0.63)	0.16 (0.06–0.25)
	ACE dropped r_C_^b^	ACE	11	18699.38	9964	–1228.62	0.96	1	.327	0.12 (0.07–0.17)	–	0.03 (–0.01–0.06)	0.40 (0.23–0.67)	–	0.10 (–0.01–0.20)
	ACE dropped r_A_ and r_C_	ACE	10	18720.89	9965	–1209.11	22.47	2	<.001						
Cognitive disorganization	Sat	–	13	18734.01	9990	–1245.99	–	–	–						
	ACE	Sat	12	18735.12	9993	–1250.88	1.11	3	.775						
	AE (retained C for tobacco)^a^	ACE	10	18735.36	9995	–1254.64	0.24	2	.886	0.17 (0.13–0.22)	–	0.03 (–0.01–0.06)	0.45 (0.31–0.64)	–	0.09 (–0.02–0.19)
	ACE dropped r_A_	ACE	11	18741.80	9994	–1246.20	6.68	1	.010						
	ACE dropped r_C_	ACE	11	18735.36	9994	–1252.64	0.24	1	.623						
	ACE dropped r_A_ and r_C_	ACE	10	18784.06	9995	–1205.94	48.94	2	<.001						
	AE dropped r_A_	ACE	9	18784.06	9996	–1207.94	48.94	3	<.001						

Note: Δdf = difference in degrees of freedom; Δ-2LL **=** log-likelihood-ratio χ^2^ test comparing each model to the base; –2LL = minus 2 log-likelihood; A = additive genetic influences; AIC = Akaike’s Information Criterion; Base = comparison model; Biva^2^=bivariate heritability; Bivc^2^ = bivariate common environments; Bive^2^ = bivariate unique environment; C = common environmental influences; df = degrees of freedom; E = unique environmental influences; EP = estimated parameters; r_A_ = genetic correlation; r_C_ = common environmental correlation; r_E_ = unique environmental correlation.

a Most parsimonious model.

b ACE models with dropped r_A_ or r_C_ indistinguishable in terms of fit and reported full ACE model results.

For tobacco use and hallucinations, ACE models dropping either r_A_ or r_C_, but not both, were not significantly worse in terms of fit compared to the full ACE model. The AIC values for the ACE models that dropped either r_A_ or r_C_ were equally low and could not be distinguished in terms of fit. These results indicated overlapping familial (genetic and/or shared environmental) influences between tobacco use and hallucinations. Thus, the full ACE model is reported.

**Figure S1:**
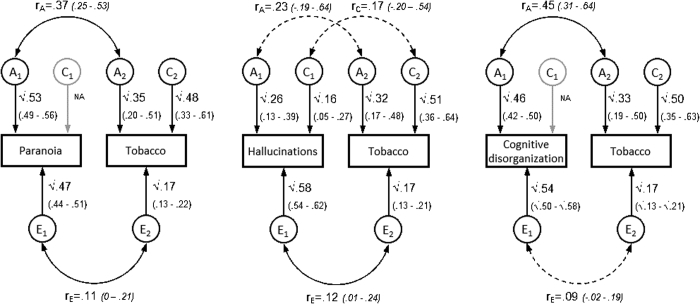
Path Diagrams and Parameter Estimates for Most Parsimonious Models: Psychotic Experiences and Tobacco Use ***Note:****Ninety-five percent CI displayed in parentheses. Dashed lines indicates nonsignificant correlations. A = additive genetic influences; C = common environmental influences; E = unique environmental influences; r*_*A*_*= genetic correlation; r*_*C*_*= common environmental correlation; r*_*E*_*= unique environmental correlation.*

**Figure S2:**
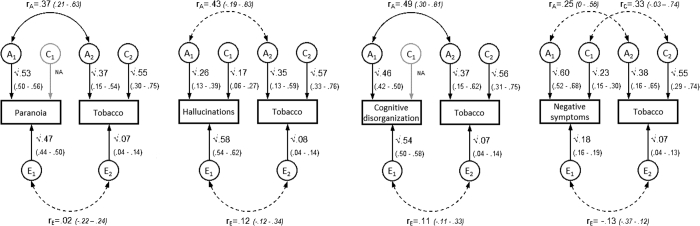
Sensitivity Analyses: Path Diagrams for Most Parsimonious Models Between Psychotic Experiences and Dichotomous Tobacco Use Variable (Nonsmokers and Smokers) ***Note:****Ninety-five percent CI displayed in parentheses. Dashed lines indicate nonsignificant correlations. Nonsmokers are defined as those who have never smoked or who have smoked less than 50 lifetime cigarettes. Smokers are those who smoked 50 or more lifetime cigarettes and who have smoked in the past year. A = additive genetic influences; C = common environmental influences; E = unique environmental influences; r*_*A*_*= genetic correlation; r*_*C*_*= common environmental correlation; r*_*E*_*= unique environmental correlation*.

Parameters from the most parsimonious models (Table 4; see Figure S1, available online) suggested significant genetic overlap between tobacco use with paranoia (r_A_ =.37) and with cognitive disorganization (r_A_ = 0.45), no shared environmental overlap, and modest overlapping unique environmental influences (r_E_ = 0.09−0.12). As a proportion of the phenotypic correlations (Table 3), genetic influences accounted for 84% of the covariance between tobacco use and paranoia and 81% between tobacco use and cognitive disorganization. The ACE model for hallucinations and tobacco indicated familial influences (A or C) accounted for 80% of the phenotypic correlation. See Table S6, available online, for bivariate statistics for full ACE models. Sensitivity analyses employing a dichotomous definition of tobacco use corroborated these findings (see Table S7, Table S8; Figure S2, available online).

## Discussion

This study investigated two separate questions about the relationship between PE and tobacco use. First, we established that associations between tobacco use and specific types of PE were present during adolescence and remained for most PE after controlling for several covariates. Second, this was the first twin study to investigate the degree to which genetic and environmental influences explain the association between tobacco use and PE. Tobacco use was associated with paranoia and cognitive disorganization largely due to overlapping genetic influences, and with hallucinations due to familial influences that may include genes and shared environment.

Our results suggest that some of the same genetic factors that influence tobacco use in adolescents also influence PE. These results may also indicate gene−environment correlations whereby the genetic factors that influence PE create environments that make tobacco use more likely, and vice versa. Although the genetic correlation could indicate a causal association between PE and tobacco use in either direction, this was beyond the scope of this paper.

Our finding that tobacco use is associated with PE in adolescents is in line with previous findings. Gage *et al.*^5^ found an association between tobacco at age 16 years and PE at age 18 after controlling for cannabis use, familial depression, maternal education, IQ, bullying, and childhood psychosocial issues. McGrath *et al.*^6^ reported that those who commenced smoking before the age of 15 years were more likely to experience hallucinations at age 21. We found similar evidence while accounting for confounders not previously considered, such as sleep disturbance and SLE.

Tobacco use predicted PE to a lesser extent after including covariates, and adjusted models explained more variation in PE than models for tobacco use only. Therefore, some of the association between tobacco use and PE was attributable to the covariates tested, most notably to sleep disturbance. Considering these results and the known etiological association between PE and sleep disturbance,^24^ future studies could explore the relationship between PE, tobacco use, and sleep disturbance. A previous TEDS study concluded that PE and cannabis use co-occurred due to shared environmental factors.^21^ In our analyses, we controlled for confounding by several other (environmental) factors, which may explain why cannabis use did not predict PE here.

We found regular smoking to be associated with paranoia, hallucinations, cognitive disorganization, and parent-rated negative symptoms, the latter two being less commonly studied compared to positive PE. The relationship between tobacco use and anhedonia diverged from this trend. Occasional smoking appears to be associated with lower anhedonia, but regular smoking, albeit not significantly, with higher anhedonia. Perhaps adolescents who experimented with tobacco, but not those who habitually smoked, were more likely to engage in pleasure-seeking behavior. Negative symptoms were parent-rated, whereas anhedonia was self-rated, which may partly account for different effect sizes between these PE types.

Another finding was that 50% of variation in adolescent tobacco use was due to common environmental influences and a third due to additive genetics. Twin studies have previously investigated the heritability of adolescent smoking.^30^ Estimates of heritability and environmental influences can change over time and are context and population specific.^39^ Anti-smoking regulations, legislation, and social attitudes toward smoking have changed rapidly over recent years. Between the early 2000s and the current study, the United Kingdom has banned smoking in public places, increased the legal age for purchasing tobacco products, introduced bold health warnings on tobacco products, restricted advertising, increased prices, and made nicotine replacement therapies more accessible.^40^

A consideration is our operationalization of the tobacco use variable. We chose a three-level rather than a binary definition to improve power and to distinguish between the effects of regular and occasional smoking, in line with previous studies.^2^, ^5^ Our twin models assume linearity among not smoking, occasional smoking, and regular smoking. The prior regression analyses showed a linear association between tobacco use with paranoia, hallucinations, and cognitive disorganization, which supports this assumption. We note that anhedonia showed a nonlinear pattern, which could be explored in future research.

Our study was not designed to assess nicotine dependency, as never-smokers, for whom susceptibility to dependency is unknown, were included in our tobacco use measure. It is known that there may be some etiological differences between smoking initiation and nicotine dependency.^41^ We also cannot rule out attrition bias, because participating families were more likely to report higher socio-economic status than nonparticipating families.

The association between PE and tobacco use is significant and modest, and findings should be viewed in this context. We could not distinguish between genetic and common environmental influences shared between tobacco use and hallucinations, likely because the phenotypic correlation was lower than for our other bivariate models. Low phenotypic correlations also meant that we could not perform bivariate models between tobacco with grandiosity, anhedonia, and parent-rated negative symptoms.

Adolescent tobacco use is modifiable risk factor. Understanding the nature of the association between PE, a possible early manifestation of psychiatric disorder in some individuals, and tobacco use is of great interest. We have contributed to this field by showing that significant associations exist between specific PE domains and tobacco use in mid-adolescence that are not fully accounted for by confounding factors. We have provided novel insights into the etiology of the covariation between some PE and tobacco use that may inform further molecular genetic studies and developmental models.

## Figures and Tables

**Table S1:** Comparison Between Participating and Nonparticipating Families

	Participating		Nonparticipating		*t* Test	*d*	*p*
	n	Mean (SD)	n	Mean (SD)			
Socio-economic status at first contact	3695	0.25 (0.98)	3722	–0.09 (0.97)	–15.09	.35	<.001
Natural mother’s age at birth of twins	3943	31.47 (4.57)	4290	30.60 (4.96)	–8.27	.18	<.001
Natural father’s age at birth of twins	3651	33.84 (5.67)	3757	33.30 (5.95)	–3.99	.09	<.001
	**n**	**%**	**n**	**%**	**χ**^**2**^	***φ***_***c***_	***p***
Ethnicity of twins					15.18	.04	<.001
White	3726	93.45%	3996	91.15%			
Other	261	6.55%	388	8.85%			
Proportion with cohabiting parents at first contact	3738	93.54%	3895	88.58%	64.29	.09	<.001
Has higher qualification							
Mothers	1172	29.72%	863	19.98%	104.72	.11	<.001
Fathers	1473	40.06%	1073	28.33%	113.78	.12	<.001
Mother’s employment status at first contact					54.48	.08	<.001
Unemployed	397	10.00%	662	15.20%			
Employed	1888	47.56%	1866	42.84%			
Staying home to look after children	1685	42.44%	1828	41.96%			
Father’s employment status at first contact					23.13	.06	<.001
Unemployed	154	4.13%	251	6.53%			
Employed	3499	93.86%	3503	91.08%			
Staying home to look after children	75	2.01%	92	2.39%			

Note: Higher qualification defined as those who have post-school qualifications (usually after the age of 18) including higher diploma/certificate, undergraduate degree, or postgraduate degrees; *d* = Cohen’s *d* where small effect *d* = 0.2, medium *d* = 0.5, and large *d* = 0.8; *φ*_*c*_ = Phi, an estimation of effect size appropriate for χ^2^ tests with small effect *φ*_*c*_ = 0.1, medium *φ*_*c*_ = 0.3, and large *φ*_*c*_ = 0.5.

**Table S2:** Frequency of Tobacco Use by Tobacco Group

Smoking frequency	Nonsmokers		Occasional Smokers (<50 Cigarettes)		Regular Smokers (≥50 Cigarettes)	
	n	%	n	%	n	%
Never tried smoking	2,789	93.43	–	–	–	–
Tried once or twice	161	5.39	294	67.43	0	0.00
Used to smoke but not now	23	0.77	53	12.16	20	10.58
<1 cigarette/wk	0	0.00	57	13.07	26	13.76
1–6 cigarettes/wk	0	0.00	6	1.38	10	5.29
>6 cigarettes/wk but not daily	0	0.00	1	0.23	7	3.70
≥1 cigarette/d	0	0.00	3	0.69	112	59.26
No information on smoking frequency	12	0.40	22	5.05	14	7.41
Total	2,985	100.00	436	100.00	189	100.00

**Table S3:** Correlation Matrix Between Psychotic Experiences Subscales and Covariates

	Paranoia	Hallucinations	Cognitive Disorganization	Grandiosity	Anhedonia	Negative Symptoms	Tobacco Use	Age	Ethnicity	Cannabis	Sleep Disturbance	Stressful Life Events	SES	Prenatal Maternal Smoking
Paranoia	1													
Hallucinations	.46§	1												
Cognitive disorganization	.42§	.44§	1											
Grandiosity	.10§	.18§	.04∗	1										
Anhedonia	.08§	.03	.01	–.19§	1									
Negative symptoms	.14§	.11§	.25§	–.02	.12§	1								
Tobacco use	.18§	.16§	.20§	.06∗	0	.08§	1							
Age	–.02	–.01	.01	.02	–.02	–.05§	.19§	1						
Ethnicity	–.01	–.09∗	–.06∗	–.20§	.02	–.01	.11∗	–.06∗	1					
Cannabis	.19§	.17§	.17§	.06∗	.04	.10§	.83§	.17§	.01	1				
Sleep disturbance	.37§	.35§	.43§	–.01	.11§	.13§	.17§	.06§	–.05	.17§	1			
Stressful life events	.16§	.17§	.13§	.16§	–.09§	.01	.32§	19§	–.13§	.39§	.14§	1		
SES	.07§	–.04∗	–.08§	–.02	.03	–.13§	–.04	–.07§	.05	0.05	.02	-.05§	1	
Prenatal maternal smoking	.05	.07§	.11§	0	–.01	.17§	.31§	.04	.08	.26§	.07∗	.10§	-39§	1

Note: Pearson correlation reported between two continuous variables. Polyserial correlations conducted between ordinal-continuous variables. Tetrachoric/polychoric correlations reported between two ordinal variables. SES = socio-economic status.

∗ *p* < .05;

§ *p* >.005.

**Table S4:** Sensitivity Analyses: Generalized Estimating Equation Models That Include Related Siblings Showing Tobacco Use as a Predictor of Psychotic Experiences

	Unadjusted Model		Adjusted Model			Unadjusted Model		Adjusted Model	
	*β*	CI	*β*	CI		*β*	CI	*β*	CI
**Paranoia**					**Grandiosity**				
Tobacco use (ref: nonsmokers)					Tobacco use (ref: nonsmokers)				
Occasional smokers	**0.32**	**0.23–.41**	**0.15**	**0.05–0.24**	Occasional smokers	–0.06	–0.15–0.03	–0.07	–0.17–0.03
Regular smokers	**0.48**	**0.33–0.64**	0.17	0–.35	Regular smokers	0.14	–0.01–0.29	0.04	–0.16–0.24
Sex			0.00	–0.06–.07	Sex			**0.27**	**0.20–0.33**
Age			–**0.10**	**–0.15 to –0.05**	Age			–0.01	–0.06–0.04
Ethnicity			–0.14	–28–0	Ethnicity			**0.26**	**0.11–0.41**
Cannabis use			0.10	–0.04–0.24	Cannabis use			0.06	–0.09–0.21
Sleep disturbance			**0.35**	**0.31–0.38**	Sleep disturbance			**–0.04**	**–0.07–0**
Significant life events			**0.09**	**0.06–0.13**	Significant life events			**0.15**	**0.12–0.19**
Socio-economic status			**0.07**	**0.03–0.10**	Socio-economic status			0.01	–0.03–0.05
Prenatal maternal smoking			0.01	–0.09–0.11	Prenatal maternal smoking			–0.03	–0.14–0.08
**Hallucinations**					**Anhedonia**				
Tobacco use (ref: nonsmokers)					Tobacco use (ref: nonsmokers)				
Occasional smokers	**0.22**	**0.13–0.32**	0.09	–0.01–0.19	Occasional smokers	0	–0.10–0.09	–0.01	–0.12–0.09
Regular smokers	**0.42**	**0.26–0.59**	**0.21**	**0.02–0.40**	Regular smokers	**0.20**	**0.02–0.38**	0.18	–0.03–0.39
Sex			–0.03	–0.09–0.04	Sex			**0.51**	**0.44–0.58**
Age			**–0.09**	**–0.14 to –0.04**	Age			–0.05	–0.10–0
Ethnicity			0.07	–0.07–0.21	Ethnicity			–0.02	–0.18–0.14
Cannabis use			0.00	–0.16–0.16	Cannabis use			0.07	–0.08–0.22
Sleep disturbance			**0.33**	**0.30–0.36**	Sleep disturbance			**0.12**	**0.08–0.16**
Significant life events			**0.11**	**0.07–0.14**	Significant life events			**–0.11**	**–0.14 to –0.07**
Socio-economic status			–0.03	–0.07–0	Socio-economic status			0.00	–0.03–0.04
Prenatal maternal smoking			0.00	–0.10–0.11	Prenatal maternal smoking			0.04	–0.07–0.16
									
**Cognitive disorganization**					**Negative symptoms**^a^				
Tobacco use (ref: nonsmokers)					Tobacco use (ref: nonsmokers)				
Occasional smokers	**0.30**	**0.20–0.39**	**0.15**	**0.05–0.24**	Occasional smokers	–0.04	–0.14–0.05	–0.07	–0.18–0.03
Regular smokers	**0.59**	**0.43–0.74**	**0.24**	**0.08–0.40**	Regular smokers	**0.52**	**0.35–0.70**	**0.28**	**0.07–0.49**
Sex			**–0.27**	**–0.33 to –0.21**	Sex			**0.17**	**0.09–0.24**
Age			**–0.05**	**–0.10 to –0.01**	Age			**–0.12**	**–0.18 to –0.06**
Ethnicity			–0.01	–0.16–.13	Ethnicity			0.05	–0.12–0.22
Cannabis use			0.05	–0.08–0.19	Cannabis use			0.10	–0.06–0.25
Sleep disturbance			**0.39**	**0.36–0.42**	Sleep disturbance			**0.14**	**0.10–0.17**
Significant life events			**0.05**	**0.02–0.08**	Significant life events			–0.03	–0.06–0.01
Socio-economic status			**–0.06**	**–0.10 to –0.03**	Socio-economic status			**–0.11**	**–0.15 to –0.07**
Prenatal maternal smoking			0.04	–0.05–0.14	Prenatal maternal smoking			**0.15**	**0.02–0.27**

Note: Both twins (using exclusion criteria as described in the Methods for bivariate twin analyses) included in Generalized Estimating Equation (GEE) models. GEE models were conducted in the *drgee* package for R. Reference group for sex: “female,” for ethnicity: “white,” for cannabis use: “no,” and for maternal smoking during pregnancy: “no.”

a Parent-rated.

**Table S5:** Univariate Liability Threshold Model Results for Tobacco Use

Model	Base	EP	Model Fit	df	AIC	Δ–2LL	Δ df	*p*	Parameters for Most Parsimonious Model		
			–2LL						A (CI)	C (CI)	E (CI)
Sat	–	10	4755.55	4894	–5032.45	–	–	–	–	–	–
Sub 1	Sat	6	4757.02	4898	–5038.98	1.47	4	.650	–	–	–
Sub 2	Sat	4	4758.86	4900	–5041.14	3.31	6	.202	–	–	–
ACE^a^	Sat	5	4758.86	4901	–5043.14	3.31	7	.860	0.32 (0.17–0.49)	0.51 (0.36–0.64)	0.17 (0.13–0.21)
AE	ACE	4	4792.97	4902	–5011.03	34.12	1	<.001	–	–	–
CE	ACE	4	4776.80	4902	–5027.20	17.94	1	<.001	–	–	–
E	ACE	3	5266.30	4903	–4539.70	507.44	2	<.001	–	–	–

Note: Δdf = difference in degrees of freedom comparing each model to the base model; Δ–2LL=log-likelihood ratio χ^2^ test comparing the –2LL fit of each model to the –2LL fit of the base model; –2LL= minus 2 log-likelihood. A = additive genetic influences; AIC = Akaike’s Information Criterion (lower values reflect a better fit); Base = comparison model (full ACE compared to saturated models and sub models compared to full ACE); C = common environmental influences; E = unique environmental influences; df = degrees of freedom; EP = number of estimated parameters; Sub 1 = Submodel 1 with equal thresholds across twin order; Sub 2 = Submodel 1 with equal thresholds across twin order and zygosity.

a Most parsimonious model.

**Table S6:** Bivariate Statistics From Full ACE Models and Best-Fitting Models for Tobacco Use and Psychotic Experiences

	Model	Bivariate Statistics From ACE and Models With Lowest AIC (95% CI)					
		Biva^2^	Bivc^2^	Bive^2^	r_A_	r_C_	r_E_
**Paranoia**	ACE	0.09 (–0.02–0.20)	0.07 (–0.01–0.17)	0.04 (0.01–0.07)	0.23 (0.01–0.52)	0.36 (–0.70–1.00)	0.13 (0.02–0.07)
	AE (retained C for tobacco)^a^	0.16 (0.11–0.21)	–	0.03 (–0.01–0.06)	0.37 (0.25–0.53)	–	0.11 (–0.01–0.21)
**Hallucinations**	ACE	0.07 (–0.05–0.19)	0.05 (–0.05–0.15)	0.04 (0–0.07)	0.25 (–0.17–0.67)	0.16 (–0.17–0.53)	0.12 (0–0.23)
	ACE dropped r_A_^b^	–	0.10 (0.06–0.14)	0.05 (0.02–0.08)	–	0.34 (0.19–0.63)	0.16 (.06–0.25)
	ACE dropped r_C_^b^	0.12 (0.07–0.17)	–	0.03 (–0.01–0.06)	0.40 (0.23–0.67)	–	0.10 (–0.01–0.20)
**Cognitive disorganization**	ACE	0.15 (0.04–0.26)	0.02 (–0.07–0.12)	0.03 (–0.01–0.06)	0.38 (0.10–0.69)	0.99 (–1–NA^c^)	0.10 (–0.02–0.21)
	AE (retained C for tobacco)^a^	0.17 (0.13–0.22)	–	0.03 (–0.01–0.06)	0.45 (0.31–0.64)	–	0.09 (–0.02–0.19)

Note: A = additive genetic influences; Biva^2^ = bivariate heritability; Bivc^2^ = bivariate common environments; Bive^2^ = bivariate unique environment; C = common environmental influences; E = unique environmental influences; r_A_ = genetic correlation; r_C_ = common environmental correlation; r_E_ = unique environmental correlation.

a Most parsimonious model.

b ACE models with dropped r_A_ or r_C_ indistinguishable in terms of fit and thus reported full ACE model results.

c Upper confidence interval not estimated likely because there was no common environmental influences on cognitive disorganization with implications for the estimate of r_C_.

**Table S7:** Sensitivity Analyses: Phenotypic Correlations and Univariate and Bivariate Twin Correlations for Dichotomous Tobacco Use Variable (Nonsmokers and Smokers)

	Tobacco			
**Phenotypic correlations**	***r***	**CI**		
Paranoia	0.17	0.11–0.23		
Hallucinations	0.16	0.10–0.22		
Cognitive disorganization	0.22	0.16–0.28		
Grandiosity and delusions	0.08	0.03–0.14		
Negative symptoms	0.22	0.16–0.28		
Anhedonia	0.08	0.03–0.14		
	**MZ**		**DZ**	
**Twin correlations**	***r***	**CI**	***r***	**CI**
Paranoia	0.53	0.49–0.56	0.30	0.25–0.35
Hallucinations	0.43	0.38–0.47	0.29	0.24–0.34
Cognitive disorganization	0.46	0.41–0.50	0.22	0.17–0.28
Negative symptoms	0.81	0.80–0.83	0.54	0.50–0.58
Tobacco	0.92	0.86–0.97	0.76	0.60–0.82
**Cross-twin cross-trait correlations (PE and tobacco)**				
Paranoia	0.16	0.09–0.26	0.10	0.02–0.17
Hallucinations	0.13	0.06–0.20	0.07	−0.01–0.14
Cognitive disorganization	0.20	0.13–0.27	0.10	0.02–0.18
Negative symptoms^a^	0.24	0.17–0.30	0.18	0.10–0.25

Note: Nonsmokers were defined as those who had never smoked or who smoked less than 50 lifetime cigarettes. Smokers were those who smoked 50 or more lifetime cigarettes and who smoked in the past year. DZ = dizygotic twins; PE = psychotic experiences.

a Parent-rated.

**Table S8:** Sensitivity Analyses: Bivariate Statistics for Psychotic Experiences and Dichotomous Tobacco Use Variable (Nonsmokers and Smokers)

	Model	Base	EP	Model fit	Df	AIC	Δ–2LL	Δ df	*p*
				–2LL					
Paranoia	Saturated model		10	15445.00	9993	–4541.00			
	ACE	Sat	11	15445.00	9994	–4543.00	0.01	1	.999
	AE (retained C for tobacco)^a^	ACE	9	15446.78	9996	–4545.22	1.78	2	.411
	ACE dropped r_A_	ACE	10	15447.58	9995	–4542.42	2.58	1	.108
	ACE dropped r_C_	ACE	10	15445.17	9995	–4544.83	0.17	1	.682
	ACE dropped r_A_ and r_C_	ACE	9	15466.39	9996	–4525.61	21.39	2	<.001
	AE dropped r_A_	ACE	8	15468.29	9997	–4525.71	23.29	3	<.001
Hallucinations	Saturated model		10	15613.03	10001	–4388.97			
	ACE	Sat	11	15613.03	10002	–4390.97	0.01	1	.999
	AE (retained C for tobacco)	ACE	9	15621.10	10004	–4386.90	8.07	2	.018
	ACE dropped r_A_	ACE	10	15614.98	10003	–4391.02	1.95	1	.162
	ACE dropped r_C_^a^	ACE	10	15613.04	10003	–4392.96	0.01	1	.929
	ACE dropped r_A_ and r_C_	ACE	9	15625.36	10004	–4382.64	12.34	2	.002
Cognitive disorganization	Saturated model		10	15583.50	9993	–4402.50			
	ACE	Sat	11	15583.62	9994	–4404.38	1.12	1	.733
	AE (retained C for tobacco) a	ACE	9	15583.63	9996	–4408.38	0.01	2	.998
	ACE dropped r_A_	ACE	10	15589.15	9995	–4400.85	5.53	1	.019
	ACE dropped r_C_	ACE	10	15583.63	9995	–4406.38	0.01	1	.944
	ACE dropped r_A_ and r_C_	ACE	9	15615.04	9996	–4376.96	31.42	2	<.001
	AE dropped r_A_	ACE	8	15615.04	9997	–4378.96	31.42	3	<.001
Negative symptoms	Saturated model		10	14113.87	10003	–5892.13			
	ACE a	Sat	11	14113.87	10004	–5894.13	0.01	1	0.999
*(parent-rated)*	AE (retained C for tobacco)	ACE	9	14144.63	10006	–5867.37	30.76	2	<.001
	ACE dropped r_A_	ACE	10	14117.12	10005	–5892.88	3.25	1	0.072
	ACE dropped r_C_	ACE	10	14117.19	10005	–5892.81	3.32	1	0.069
	ACE dropped r_A_ and r_C_	ACE	9	14162.75	10006	–5849.25	48.88	2	<.001

Note: Saturated models constrained means, thresholds and phenotypic correlations across twin order. Nonsmokers defined as those who had never smoked or who smoked less than 50 lifetime cigarettes. Smokers were those who smoked 50 or more lifetime cigarettes and who smoked in the past year. –2LL = minus 2 log-likelihood; Δdf = difference in degrees of freedom compared to the base model; Δ–2LL=log-likelihood ratio chi-square test comparing the –2LL of each model to the –2LL fit of the base model; A = additive genetic influences; Base = comparison model; AIC = Akaike's Information Criterion (lower values reflect a more parsimonious and therefore preferred fit); C = common environmental influences; df = degrees of freedom; E = unique environmental influences; EP = estimated parameters; r_A_ = genetic correlation; r_C_ = common environmental correlation; r_E_ = unique environmental correlation

a Most parsimonious model.
